# An unusual granulomatous tinea due to *Nannizzia incurvata* in Germany without evidence of an acquisition from outside Europe and reduced itraconazole susceptibility

**DOI:** 10.1016/j.mmcr.2026.100769

**Published:** 2026-02-02

**Authors:** Elisabeth Anke Schuldt, Karen Voss, Thomas Schwarz, Stephan Weidinger, Dora Violetta Stölzl, Jochen Brasch

**Affiliations:** Department of Dermatology, Venereology and Allergology, University Hospital Schleswig-Holstein, Campus Kiel, Kiel, Germany

**Keywords:** Dermatophytes, Histopathology, Diagnostics, Antifungal susceptibility, Epidemiology

## Abstract

*Nannizzia (N.) incurvata* is a rare geo- and zoophilic dermatophyte with human infections only sporadically reported from Europe. We describe an atypical skin infection with granulomas from Germany confirmed by culture, PCR, and histopathology. A reduced susceptibility to itraconazole (unreported for *N. incurvata* so far) prompted systemic treatment with terbinafine that resulted in complete healing. This case highlights the need for precise species identification, susceptibility testing, and awareness of emerging dermatophytes in Europe.

**2012 Elsevier Ltd:**

All rights reserved.

## 1 Introduction

*Nannizzia (N.) incurvata* is a geo- and zoophilic fungus closely related to *N. gypsea*. It can infect animals but can cause skin infections in humans on rare occasions as well. Human *N. incurvata-*infections have been reported from Asia but up to now only very few infections with *N. incurvata* have been communicated from Europe. To our knowledge, all previously published European cases had a connection to Asian countries. Our case is noteworthy because, firstly, it showed atypical clinical features with a granulomatous tissue reaction; secondly, a German woman with no relation to a non-European country was affected; and, thirdly, the isolated *N. incurvata-*strain unexpectedly had a reduced itraconazole susceptibility.

## 2 Case presentation

A 54-year-old woman in good general health was referred to our outpatient clinic by her internist who proposed a skin biopsy in order to verify a diagnosis of chronic discoid lupus erythematosus (CDLE). A preceding treatment with a cream containing fusidic acid and betamethasone had not resulted in any improvement. The patient complained of progressive itching macules on her face and arms that had been present for approximately two months. At the time these lesions had emerged, she had been on vacation in a nature reserve on Sardinia, Italy. She also reported that about one year earlier, after a holiday on Ibiza, she had noted similar skin lesions on her right upper arm which had elsewhere been judged as tinea (without mycological diagnostics) and had healed after several weeks of topical antimycotic treatment. An extended history revealed that her husband had recently developed two similar skin lesions, which had healed spontaneously within a short time. The patient kept chicken and cats on her property with no obvious abnormalities of plumage or fur.

### 2.1 Skin findings

**Fig. 1:**
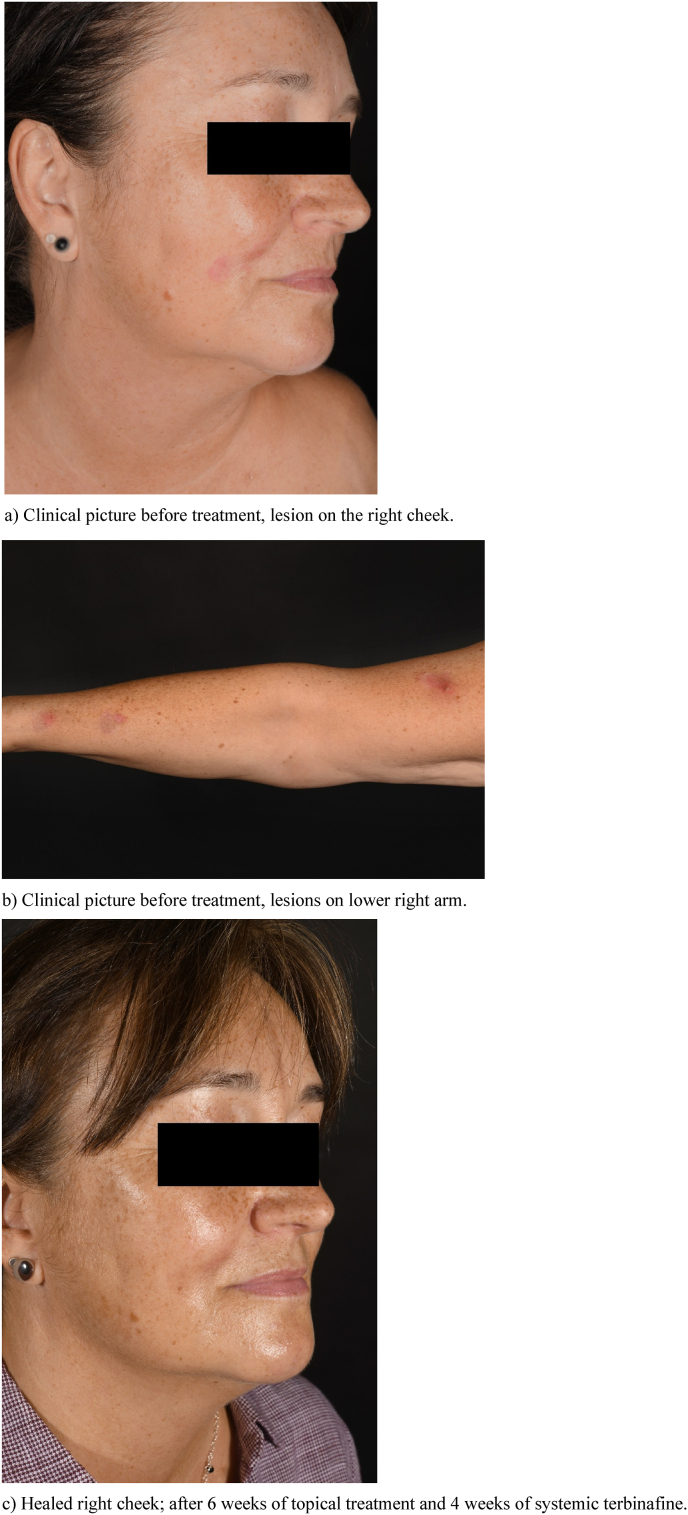
Clinical picture.

On the right cheek (Fig. 1a) and on both lower arms (Fig. 1b) and thighs there were distinctly outlined violaceous-erythematous plaques with diameters of 1–3 cm, elevated borders and very little scaling. On the right arm sparse flat small papules were apparent (Fig. 1b). No pustules were visible.

### 2.2 Diagnostics

**Fig. 2:**
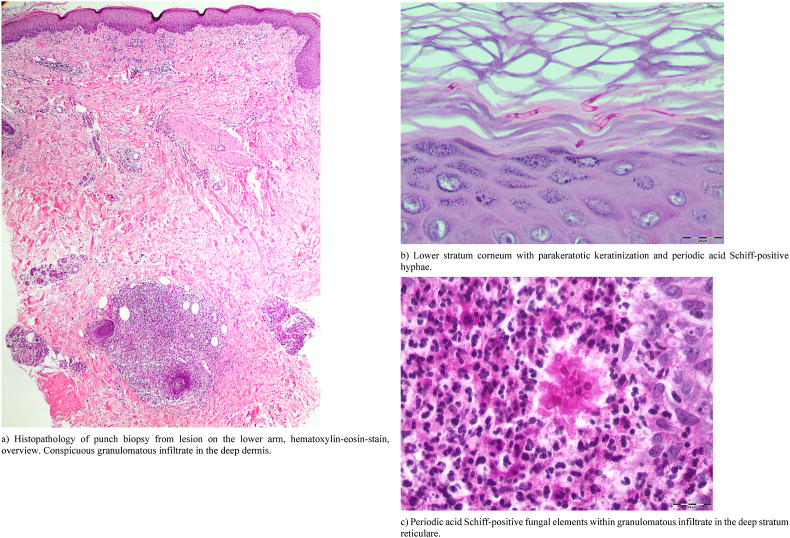
Histopathology.

**Fig. 3:**
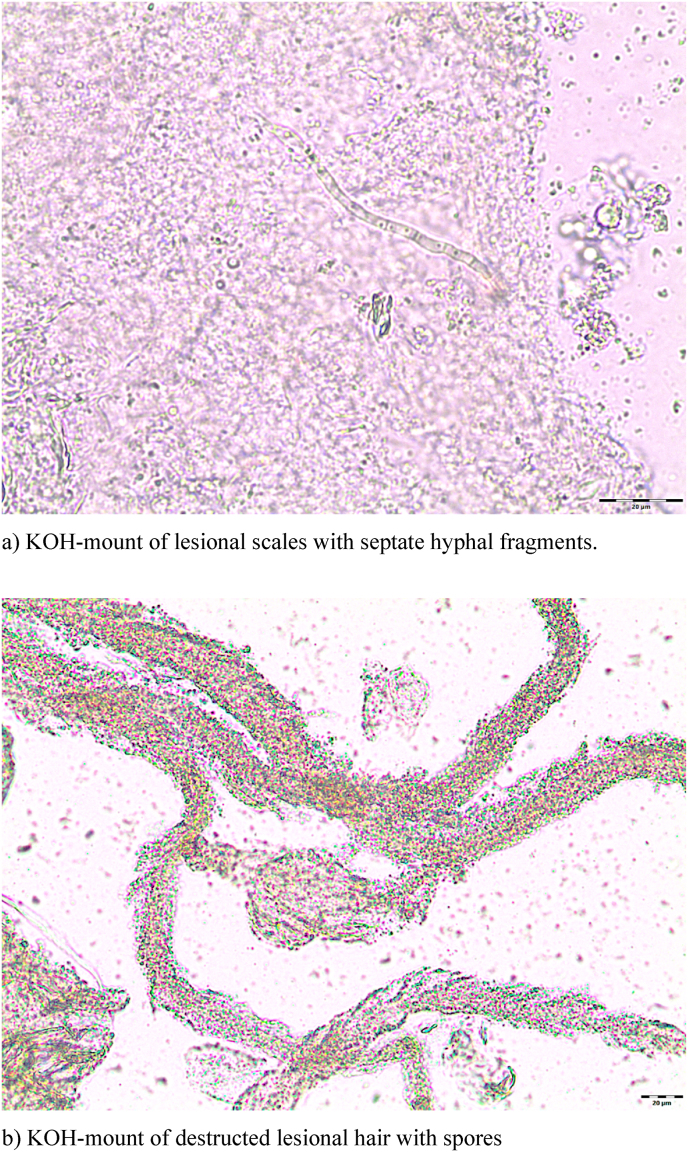
KOH-mounts.

**Fig. 4:**
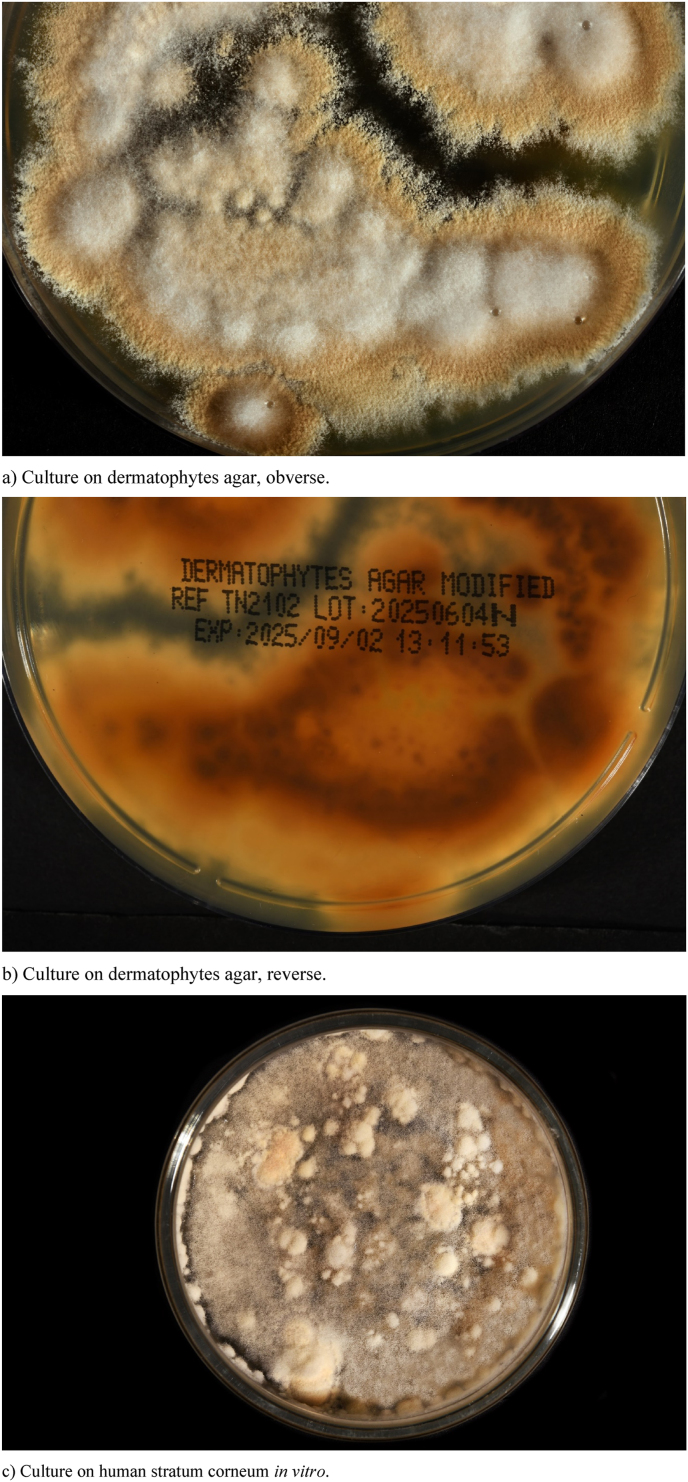
Cultures of *Nannizzia incurvate*.

**Fig. 5:**
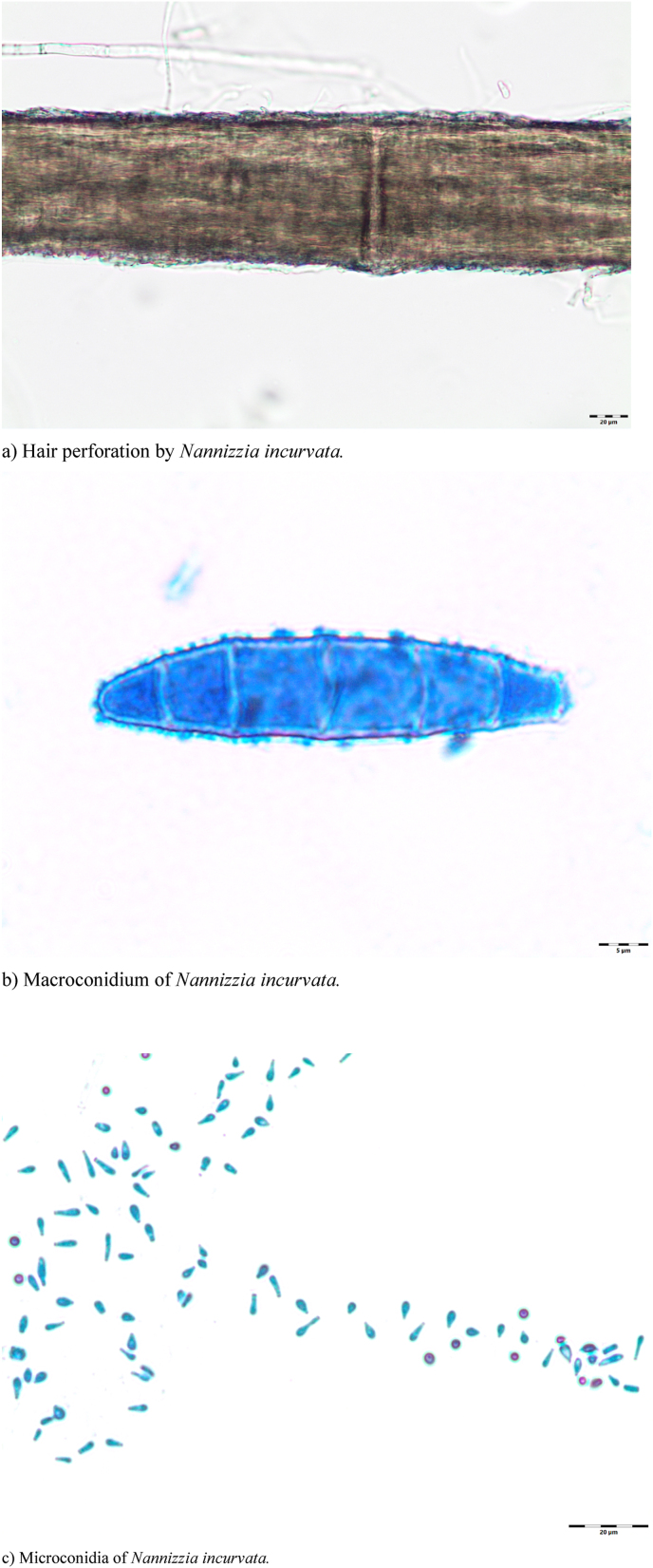
Microscopy of *Nannizzia incurvata*.

Because a diagnosis of CDLE had been suggested, a punch biopsy was taken from a lesion on her right lower arm at the initial visit. The histopathology revealed parakeratotic keratinization in the lower stratum corneum (Fig. 2a and b). Focally, some scattered intraepidermal leukocytes were present. A sparse mixed infiltrate of mainly mononuclear cells plus some neutrophils was found within the upper stratum reticulare with perivascular accentuation (hematoxylin-eosin (HE)-stain) (Fig. 2a). Dense granulomatous aggregates of mononuclear cells with small neutrophilic abscesses were conspicuous in the deep stratum reticulare (Fig. 2a). Periodic acid Schiff (PAS)-stains demonstrated intracorneal hyphal elements (Fig. 2b) and rounded PAS-positive structures compatible with fungal elements within these granulomas (Fig. 2c). These findings proved a superficial fungal skin associated with a granulomatous dermal mycotic component. Therefore, formalin-fixed slides were subsequently used for PCR (EUROArray Dermatomycosis®-assay) as described earlier [1]. As a result, *N. incurvata* was identified in the biopsied tissue. Furthermore, fresh lesional scales were collected for mycological diagnostics. KOH-mounts showed short, hyaline, septate hyphal fragments (Fig. 3a) and destructed hairs enveloped and presumably interspersed with small spores (Fig. 3b). PCR screening (EUROArray Dermatomycosis®) of the collected scales confirmed *N. incurvata.* On dermatophytes agar with cycloheximide and on Sabouraud agar powdery white colonies grew within days, later developing a light brown pigmentation towards the edges (Fig. 4 a). The reverse was yellowish to light brown-orange in the centre (Fig. 4b). The conventional mycological characterization was then extended with methods described previously [2]. The strain grew rapidly on human stratum corneum in vitro (Fig. 4c). No growth was observed within 6 weeks on dermatophytes agar at 4 °C. The urease test was positive, likewise the hair perforation test (Fig. 5a). Microscopically multiple pluricellular macroconidia were detected in slide cultures with moderately thick and rough walls (Fig. 5 b) plus plentiful clavate, thin-walled microconidia attached alongside hyphae (Fig. 5c). All of these findings are consistent with *N. incurvata.*

A semi-quantitative antifungal susceptibility testing [2] showed no growth of the isolate with 0.15 mg/L terbinafine in RPMI-agar (Gibco RPMI Medium 1650, Life Technologies Ltd., Paisley, UK) within 14 days. However, with 1.0 mg/L itraconazole in RPMI-agar its growth was only partially suppressed with culture diameters still reaching as much as 20 mm after 14 days - as compared to 60 mm in controls without itraconazole. By comparison, most common dermatophytes like *Trichophyton rubrum* are completely inhibited by 1.0 mg/L itraconazole in this assay (and by 0.15 mg/L terbinafine as well).

*Our strain is* preserved in the public German Collection of Microorganisms and Cell Cultures, Braunschweig, Germany; *DSM No.* 121183.

### 2.3 Clinical course

After the diagnosis of tinea had been established treatment was started topically with ciclopiroxolamine cream. Two weeks later, in accordance with the results of the tests with antimycotics, terbinafine 250 mg/day p. o. was added. Under this combination the skin lesions improved very rapidly. Terbinafine was well tolerated, and four weeks after start of oral terbinafine only a postinflammatory hyperpigmentation had remained at the affected sites (Fig. 1c). Since all scaling had resolved at this point of time a mycological control was not feasible then and clinical healing was determined. Systemic terbinafine was discontinued after four weeks and we only recommended to apply ciclopiroxolamine cream for an additional four weeks as a precaution. Finally, 12 weeks after cessation of any therapy the patient reported to have remained symptom-free and that the residual hyperpigmentation was fading. The patient’s cat had been considered for a fungal reservoir because presumably this was the patient’s second fungal infection. Although the cat showed no fur abnormalities, it was preventively treated topically with an antifungal agent by a veterinarian.

## 3 Discussion

*N. incurvata* was first described by Stockdale in 1961 under the name *Microsporum incurvatum*. In 1963, it was allocated to the so-called *N. gypsea* complex, which includes, among others, *N. gypsea*, *N. incurvata*, and *N. fulva* [3]. In total, the genus *Nannizzia* currently comprises 13 geo- and zoophilic species [4].

*N. incurvata* is a mainly geophilic dermatophyte but can cause favus in cats and dogs [5]. Furthermore, *N. incurvata* can be pathogenic to humans under favorable conditions. Reported human infections include tinea corporis [6], favic tinea capitis with subsequent hair loss, and tinea faciei [7]. In contrast to infections with the closely related *N. gypsea*, which is distributed worldwide, human infections with *N. incurvata* are rare. They occur most frequently in Southeast Asia, particularly in Vietnam and Sri Lanka [7,8]. In China, two cases of scrotal tinea caused by *N. incurvata* have been described; otherwise, the predominant cause of scrotal tinea in China is *N. gypsea* [9]. Case reports of *N. incurvata-*infections in humans are exceptional in Europe*;* only two infections in Germany have been reported: a traveler returning from Indonesia 2024 and a Vietnamese female who returned from a visit to her homeland 2021 [6,7]. In our case, there was no clue for a direct or indirect contact of the patient with a possible source of *N. incurvata* in Asia.

The hardly inflammatory discoid lesions in sun-exposed skin had prompted the referring internist to suspect CDLE. This lack of a marked inflammatory response is in fact unusual for human *N. incurvata-*infections [7,10]. Therefore, the detection of granulomatous infiltrates with fungal elements within the deep stratum reticulare was surprising and to the best of our knowledge, such histopathological findings in human *N. incurvata-*infections have not been communicated before. A recent investigation of cat favus due to *N. incurvata* described lesional histopathological features but these included no dermal invasion by fungi or formation of granulomas [11]. Our finding now shows that the clinical appearance of a *N. incurvata-*infection can be pretty bland and deceptive despite of a marked involvement of deeper skin layers. This discrepancy can be relevant because in contrast to strictly superficial tinea deep granulomatous fungal infections necessitate systemic treatment.

Data on antifungal susceptibility of *N. incurvata* are very limited up to now. In our case, terbinafine was chosen for systemic treatment due to the reduced susceptibility to itraconazole in our test. This attenuated effectiveness of itraconazole came as a surprise because a reduced responsiveness to itraconazole of *N. incurvata* had not been reported before. A Vietnamese study in fact reported normal susceptibility to itraconazole (as well as to voriconazole, clotrimazole, miconazole, and terbinafine) in 12 isolates [8] but interestingly these authors also observed 100% resistance to fluconazole [8]. In our test procedure ordinary strains of common dermatophyte species (*Trichophyton (T.) rubrum*, *T. mentagrophytes* etc.) are completely inhibited by 1.0 mg/L itraconazole. Since further data on antifungal susceptibility of *N. incurvata* are lacking, we suggest to test future isolates for possible resistance to antimycotics. In addition to a systemic therapy, a concomitant topical antimycotic treatment is generally recommended in cases of tinea.

Since up to now there is no evidence of human-to-human transmission of *N. incurvata,* contact tracing and treatment of contacts are usually not required [7]. The route of transmission has not been fully clarified. Because *N. incurvata* is primarily geophilic, infection through contact with contaminated soil is possible. Bottom-dwelling animals may also be carriers without or with visible lesions. A Brazilian study identified *N. incurvata* as a causative agent of dermatophytosis in dogs and cats [5]. Considering that our patient’s husband reported previous transient skin lesions similar to those of the patient and because the patient herself probably had experienced an earlier infection as well it is our speculation that in our case a cat may have acted as a potential reservoir of *N. incurvata.* This might have been the patient’s own cat or some stray cat she petted during her stays on Ibiza or Sardinia. The lacking growth of our strain at 4 °C is compatible with a distribution of this species in the soil of warm climate zones and with fur colonization of mammals.

Our case demonstrates that *N. incurvata* should be considered as a possible cause of dermatomycosis not only in Asia but also in Europe. It also reveals that a *N. incurvata-*infection can present with an atypical clinical picture including a granulomatous tissue reaction and that reduced susceptibility to itraconazole may occur in this species. We suggest to check mammals with soil contact for colonization with *N. incurvata* in the surroundings of a *N. incurvata*-infection.

## CRediT authorship contribution statement

**Elisabeth Anke Schuldt:** Conceptualization, Writing – original draft. **Karen Voss:** Investigation. **Thomas Schwarz:** Writing – review & editing. **Stephan Weidinger:** Writing – review & editing. **Dora Violetta Stölzl:** Methodology, Visualization, Writing – review & editing. **Jochen Brasch:** Resources, Writing – review & editing, Supervision.

## Conflict of interest

ES has received honoraria for lectures or publications from Sanofi, Regeneron, LEO Pharma and Pfizer. DS has received honoraria for lectures and/or scientific advisory activities from Sanofi, Regeneron, LEO Pharma, AbbVie, Almirall, Pfizer, and Novartis. SW has received consulting fees, honoraria, grant support, and/or lecture fees from AbbVie, Almirall, Apogee, Astria, Boehringer, Eli Lilly, Galderma, GSK, Incyte, Leo Pharma, Pfizer, Regeneron Pharmaceuticals, Sanofi, and UCB.
